# Exploring the multi-dimensional coordination relationship between population urbanization and land urbanization based on the MDCE model: A case study of the Yangtze River Economic Belt, China

**DOI:** 10.1371/journal.pone.0253898

**Published:** 2021-06-30

**Authors:** Ling Shan, Yuehua Jiang, Cuicui Liu, Yufei Wang, Guanghong Zhang, Xufeng Cui, Fei Li

**Affiliations:** 1 School of Business Administration, Zhongnan University of Economics and Law, Wuhan, Hubei, China; 2 School of Information and Safety Engineering, Zhongnan University of Economics and Law, Wuhan, Hubei, China; Northeastern University (Shenyang China), CHINA

## Abstract

The rapid development of urbanization has had a dramatic impact on the economy, society and environment in China. In this context, the coordination relationship between population urbanization and land urbanization is essential for achieving sustainable urbanization. Based on the statistical data from 2007–2017 in the Yangtze River Economic Belt (YEB), this paper established the multi-dimensional coordination evaluation (MDCE) model by using the speed coordination evaluation (SCE) model, the level consistency evaluation (LCE) model, the entropy method and the space matching evaluation (SME) model to evaluate the coordination relationship between population urbanization and land urbanization from the speed-level-space perspective. The results showed that from 2007 to 2017: 1) the development speed of population urbanization and land urbanization in the YEB were more and more coordinated, and the speed of population urbanization lagged behind that of land urbanization. In addition, the overall development speed of the 11 provinces declined, and most of them were characterized by excessive development of land urbanization. 2) the development level of population urbanization and land urbanization in the YEB were all high, but the development level of population urbanization was lower than that of land urbanization. Further, the development level of the 11 provinces remained stable and high, and continuously improved. 3) the space matching of population urbanization and land urbanization in the YEB had a high degree of coordination, and the space matching degree of population urbanization was higher than that of land urbanization. Moreover, the space matching of most provinces in the region had declined, but the change was small. Finally, this paper proposes the policy recommendations on the coordinated development of population and land urbanization at the institutional, market and management levels to achieve coordinated and sustainable urbanization.

## 1. Introduction

With the accelerated development of industrialization, the global mobility of capital and labor force is increasing, and a large-scale urbanization movement has been launched in the world. China is one of the countries with the fastest urbanization development [1]. Since the reform and opening-up policy was implemented, China has made remarkable achievements in the development of urbanization, the number of urban population has increased from 172.45 million in 1978 to 848.43 million in 2019. The urbanization rate has increased from 17.92% in 1978 to 60.60% in 2019 [2]. However, China’s rapid urbanization has also brought some severe problems, such as overcrowding [3], urban land disorderly expansion [4–6], loss of cultivated land [7, 8], loss of population [9] and environmental degradation [10–14], etc. It can be seen that sustainable urbanization is an effective way to promote the sustainable development of China’s urban area and it plays an important role in the overall development of the society.

Urbanization mainly includes population urbanization and land urbanization. Population urbanization is the core of urbanization [15], land urbanization is the carrier of urbanization [16], and the development speed of land urbanization is faster than population urbanization [17, 18]. In this context, it is necessary to study the relationship between population urbanization and land urbanization. However, most studies discussed the coupling coordination relationship between population and land urbanization based on a single dimension of development level, lacking multi-dimensional perspective [19–21].

Besides, in recent years, the Yangtze River Economic Belt (YEB) has become a major platform for driving China’s rapid economic growth and participating in international economic cooperation and competition. In 2019, the GDP of YEB is 45780.5 billion yuan, accounting for 46.2% of the whole country. The population of the region is 602.058 million people, accounting for 42.9% of the whole country, and the area is about 2.05 million square kilometers, accounting for 21.4% of the whole country [22], which brings great pressure to the limited land resources. How to coordinate the population urbanization and land urbanization and get rid of the "rash" urbanization development has become a problem that should be considered in the YEB.

Therefore, based on the statistical data from 2007–2017, this paper takes the YEB as the research area, and constructs a multi-dimensional coordination evaluation (MDCE) model to evaluatethe the spatial-temporal pattern evolution and coupling coordination relationship between population urbanization and land urbanization from the speed-level-space perspective. In addition, the goals of this study are: i) to explore the relationship between population urbanization and land urbanization from the speed-level-space perspective based on the MDCE model; ii) to analyze characteristics of spatiotemporal pattern evolution of the multi-dimensional coordination relationship between population urbanization and land urbanization; iii) to promote the coordinated development of population-land urbanization in the YEB, achieve sustainable and healthy development of the economy and society, and provide relevant policy support. The realization of these goals will promote the coordinated development of population urbanization and land urbanization in the YEB, and help urban planners and policy makers to solve some problems caused by the rapid development of urbanization, and then put forward differentiated sustainable development strategies of urbanization.

## 2. Literature review

Nowadays, a copious literature has studied the issues related to urbanization, such as income inequality [23, 24], economic growth issues [25–27], environmental issues [28–31], hoping to promote the coordinated development of cities and achieve sustainable urbanization. In addition, various empirical studies have explored the impact of urbanization in different parts of the world. Cobbinah et al. compiled relevant literatures about the impacts of rapid urbanization on sustainable development of Africa, and found that the nature of urbanization had some positive implications for socio-economic development and management [32]. In the relevant theoretical discussion, Jain used a combination of statistical analysis and field survey to clarify the process and characteristics of urbanization in India. He advocated for shifting traditional master planning to achieve integrated development of infrastructure [33]. Muñoz et al. investigated the impact of Austria’s urbanization on carbon dioxide emissions and found that due to more compact cities, Austria’s urbanization may represent a relative reduction of emissions in the future [34]. Liu et al. estimated the relationship between urbanization and atmospheric environment security in Jinan City from 1996 to 2004 on the basis of the theory of Environmental Kuznets Curves (EKC) [35].

The connotation of urbanization includes population urbanization and land urbanization, and sustainable urbanization refers to be the coordinated development of population urbanization and land urbanization. Population urbanization refers to the process of transferring the population from the rural area to the urban in order to integrate the rural population into the city. The essence of population urbanization is the process of transferring rural population, funds, resources and materials to cities, as well as changes in living and producing styles, values and behavior [36]. Population urbanization can improve living standards and consumption levels of the rural population, and guarantee the basic infrastructure and social public services [37]. Land urbanization refers to the change of land ownership and land use in the process of urbanization. It contains not only the increase of the urban land area, but also the change of the land use pattern and structure [15]. Land urbanization turns the cultivated land into construction land, expands the urban land areas and increases the urban built-up areas [37]. Hence, the coordination of population urbanization and land urbanization is conducive to the healthy and sustainable urbanization.

Recently, scholars’ research on population urbanization mainly focuses on three aspects: spatial distribution [38], influencing factors [39], and development path [40]. And scholars’ research on land urbanization has focused on spatiotemporal pattern evolution [41, 42], driving forces [43] and urban land expansion [44, 45]. Nevertheless, most scholars have researched the coordinated development relationship between population urbanization and land urbanization. For example, Lin et al. analyzed the interactive relationship between population urbanization and land urbanization in Chongqing, China, from 1998 to 2016. The results of the study showed that the land population was faster than population urbanization, but the improvement in the quality of the population urbanization development was greater than that of land population [46]. Han et al. analyzed the coordination degree, spatial heterogeneity, and spatiotemporal evolution of urbanization of 10 cities in Ha-Chang urban agglomeration in China from 2000 to 2014. They found that the quality of population and land urbanization was increasing gradually, while the degree of coordination between population and land urbanization rate was declining [47]. Yin et al. and Tan et al. separately evaluated in all 644 cities and 31 provinces of China about the coordination relationship between population urbanization and land urbanization. It showed that except a few cities and provinces, the population urbanization development level of most cities in China lagged behind the land urbanization development level [48, 49]. According to these literature, the level of population urbanization and land urbanization has uncoordinated problems in current China, and the overall situation of land urbanization is over-developed. The lag of population urbanization creates problems such as idle urban land or crowed central urban areas, and relates to unreasonable resource consumption. Therefore, it is worth noting that developing a sound and interactive relationship, not only should we realize the coordination of the development speed and development level, but also should we pay attention to the development space. Otherwise, even if the population and land urbanization achieve the coordinated development, there will still be spatial imbalance.

Moreover, various studies have been conducted using different methods to explore the uncoordinated development of population urbanization and land urbanization, such as the coupling coordination degree model [50], the extended STIRPAT model [51], the TOPSIS comprehensive evaluation method [52], and the logistic regression model [53]. Among them, the coupling coordination degree model is the most widely used. Originating from physics, coupling refers to a measure of the mutual dependence of two entities. The coupling coordination degree model is used to analyze the coordinated development level between things, and the coupling relationship refers to the interaction between the two things. Thus, the coupling coordination degree model can effectively explain the coordinated development relationship between population urbanization and land urbanization.

Generally speaking, the research on the relationship of population-land urbanization is mainly concentrated at the provincial and municipal levels [54, 55], while the research on urban agglomeration is less. Especially urban agglomeration has become an important form of urbanization, and it is necessary to explore the urbanization of urban agglomeration. Meanwhile, most scholars evaluate the coordinated development of population-land urbanization from a single dimension, only investigating the coordination degree of their development level. There are few research from a multi-dimensional perspective to research the coordinated development. Therefore, this paper aims to evaluate the coordinated relationship between population urbanization and land urbanization of the YEB by using the MDCE model from the perspective of speed-level-space. Then, the corresponding policies and recommendations for the coordinated development of population-land urbanization are putting forward.

## 3. Materials and methods

### 3.1. Study area

The Yangtze River is the largest river in China and the third longest river in the world. The main stream is 6300km long and the drainage area is 1.8 million square kilometers. The Yangtze River Basin refers to the vast area that the main stream and tributaries of the Yangtze River flow through, with a total drainage area of about 1.8 million square kilometers, accounting for 18.8% of the land area of the country. After thousands of years of development and construction, the Yangtze River Basin has become one of the most developed areas in China in terms of agriculture, industry, commerce, culture, education, science and technology. The Yangtze River economic belt (YEB) is the most developed area in the Yangtze River Basin and one of the high-density economic corridors in China.

**Fig 1:**
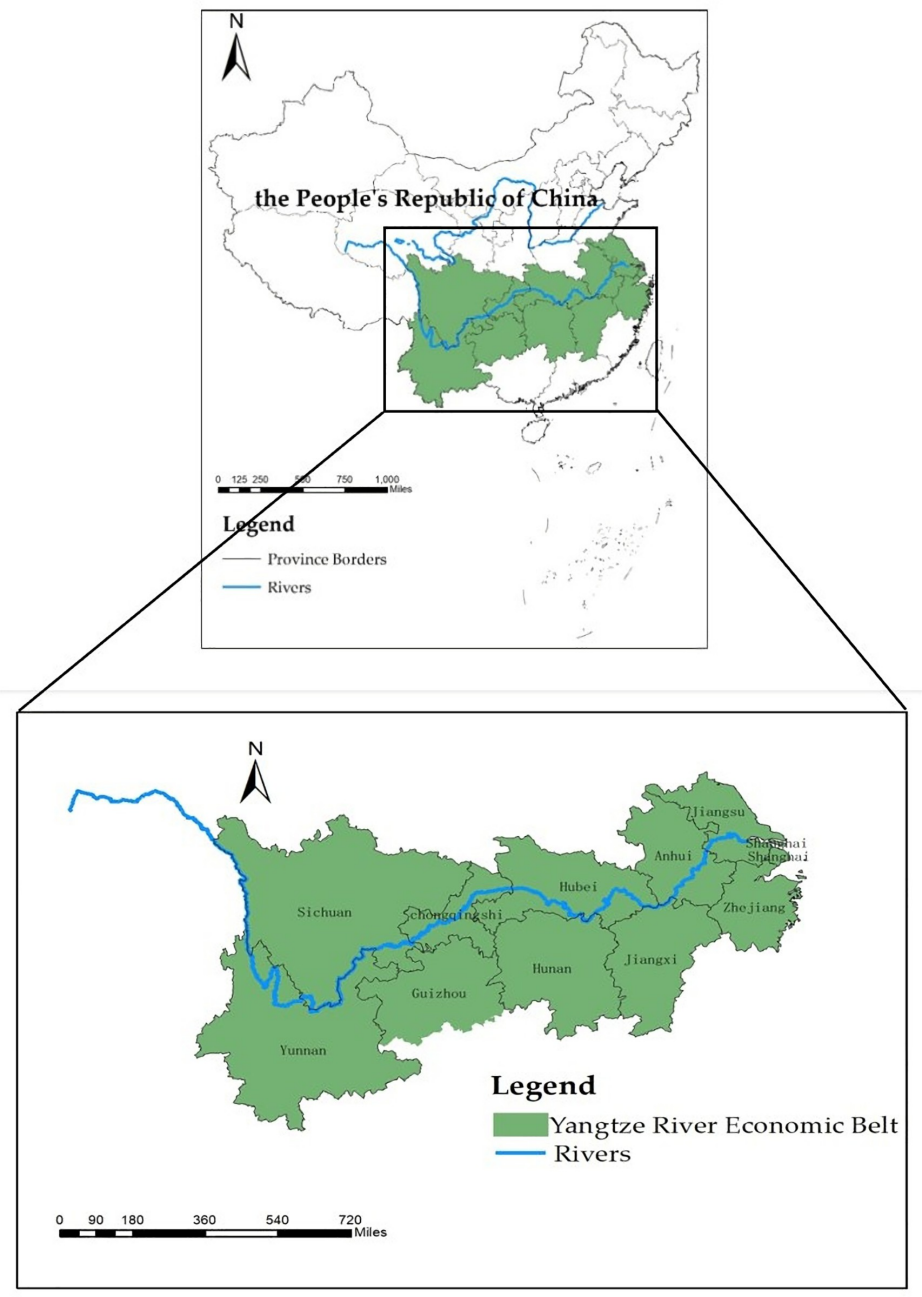
Map of the study area. (Quoted from the Tianditu map. Source of base map: the open source map data service provided by the National Platform for Common GeoSpatial Information Services (https://www.tianditu.gov.cn).

With the rapid development of economy and society, the contradiction between human and land in the YEB is becoming increasingly acute, so this paper takes the YEB as the study area. The YEB covers 9 provinces and 2 municipalities, namely, Shanghai, Jiangsu, Zhejiang, Anhui, Jiangxi, Hubei, Hunan, Chongqing, Sichuan, Guizhou and Yunnan [56] (Fig 1). The YEB is divided into three regions according to the upper, middle and lower reaches of the Yangtze River: the lower reaches include Shanghai, Jiangsu, Zhejiang and Anhui; the middle reaches include Jiangxi, Hubei and Hunan; and the upper reaches include Chongqing, Sichuan, Guizhou and Yunnan. In general, the urbanization rate of the YEB is high, the development speed is fast, and the regional development is unbalanced. In 2019, the average urbanization rate of provinces and cities in the YEB is 61.7%, which is higher than the national average level (60.60%). The urbanization rate of Jiangsu, Zhejiang and Shanghai in the lower reaches is more than 70%, with the highest in Shanghai being 88.10%; Hubei in the middle reaches and Chongqing in the upper reaches are also more than 60%, but less than 70%, which are at the end of the rapid development period; the urbanization rate of Yunnan and Guizhou in the upper reaches of the Yangtze River is lower, less than 50% [22].

The YEB spans three major regions of China’s eastern, central, and western regions, covering an area of about 2.0523 million square kilometers, accounting for 21.4% of China’s total area, with a population and GDP exceeding 40% of the country [57]. It is an inland river economic belt with global influence, a coordinated development zone for interaction and cooperation, and a comprehensive development along the coast and along the river [58]. Moreover, in 2014, the State Council issued *the guiding opinions on promoting the development of the Yangtze River Economic Belt relying on the golden waterway*, which officially proposed for the first time to promote the coordinated development of the upper, middle and lower reaches of the Yangtze River and build the YEB. Subsequently, in 2016, Chinese Government issued the *Outline of the Development Plan for the Yangtze River Economic Belt*, which is to promote the development of the YEB as a national strategy, and indicated that the YEB had become a strong support for the sustainable and healthy development of China’s economy. However, the development of the YEB is facing many difficulties and problems to be solved urgently, such as the severe situation of ecological environment, the prominent imbalance of regional development, the arduous task of industrial transformation and upgrading, and the imperfect regional cooperation mechanism. Considering that the region has the characteristics of strong comprehensive strength, wide coverage and huge regional differences, it is of great significance to explore the characteristics of urbanization and the interactive relationship between population and land urbanization in the YEB.

### 3.2. Data sources

Based on the speed coordination, level consistency and space matching of the population-land urbanization, this paper evaluated the multi-dimensional coordinated development of population-land urbanization in the YEB from 2007 to 2017. The relevant research data was basically collected from the China Statistical Yearbooks (2008–2018) [59], China City Statistical Yearbook (2008–2018) [60], and the related provincial statistical yearbooks (2008–2018). This paper used 2007 as the base period for comparative research. Some indicators that cannot be directly calculated were calculated mathematically. In addition, price indexes were deflated in the study of time series in order to eliminate the impact of inflation, price increases and other factors on the economy.

### 3.3. Methods

In this paper, the multi-dimensional coordination evaluation (MDCE) refers to be the analysis of the coordinated development of population-land urbanization from multiple dimensions and different perspectives (i.e., speed coordination, level consistency and space matching of the population-land urbanization). If the two are in a balanced state, the development speed and development level of population urbanization will be consistent with the development speed and development level of land urbanization in a region, achieving urban-rural balanced development and industrial transformation and upgrading. If the two were in an unbalanced state (e.g., the speed and area of land expansion are too fast), it will lead land resources to be inefficiently used.

The speed coordination refers to be the coordination degree between the growth rate of population urbanization and the growth rate of land urbanization in an area within a certain period of time. The excessively rapid population urbanization process will lead to urban congestion, environmental degradation and uneven distribution of urban resources, while the excessively rapid land urbanization process will lead to disorderly expansion of urban land and inefficient use of land resources. The level consistency refers to be the coordination degree between the development level of population urbanization and the development level of land urbanization in an area within a certain period of time. If the development level of population urbanization is lower than that of land urbanization, it will cause land resources disorderly developed and empty and ghost towns. If the development level of population urbanization is higher than that of land urbanization, it will cause urban congestion and reduce the living standards of urban residents. The space matching refers to be the coordination degree between the space matching degree of urban population and the space matching degree of land urbanization in an area within a certain period of time. If the space matching degree of land urbanization is lower than that of population urbanization, it will lead to excessive consumption of important resources (e.g., cultivated land and water resources) and environmental pollution. If the space matching degree of population urbanization is lower than that of land urbanization, it will cause extensive land use.

**Fig 2:**
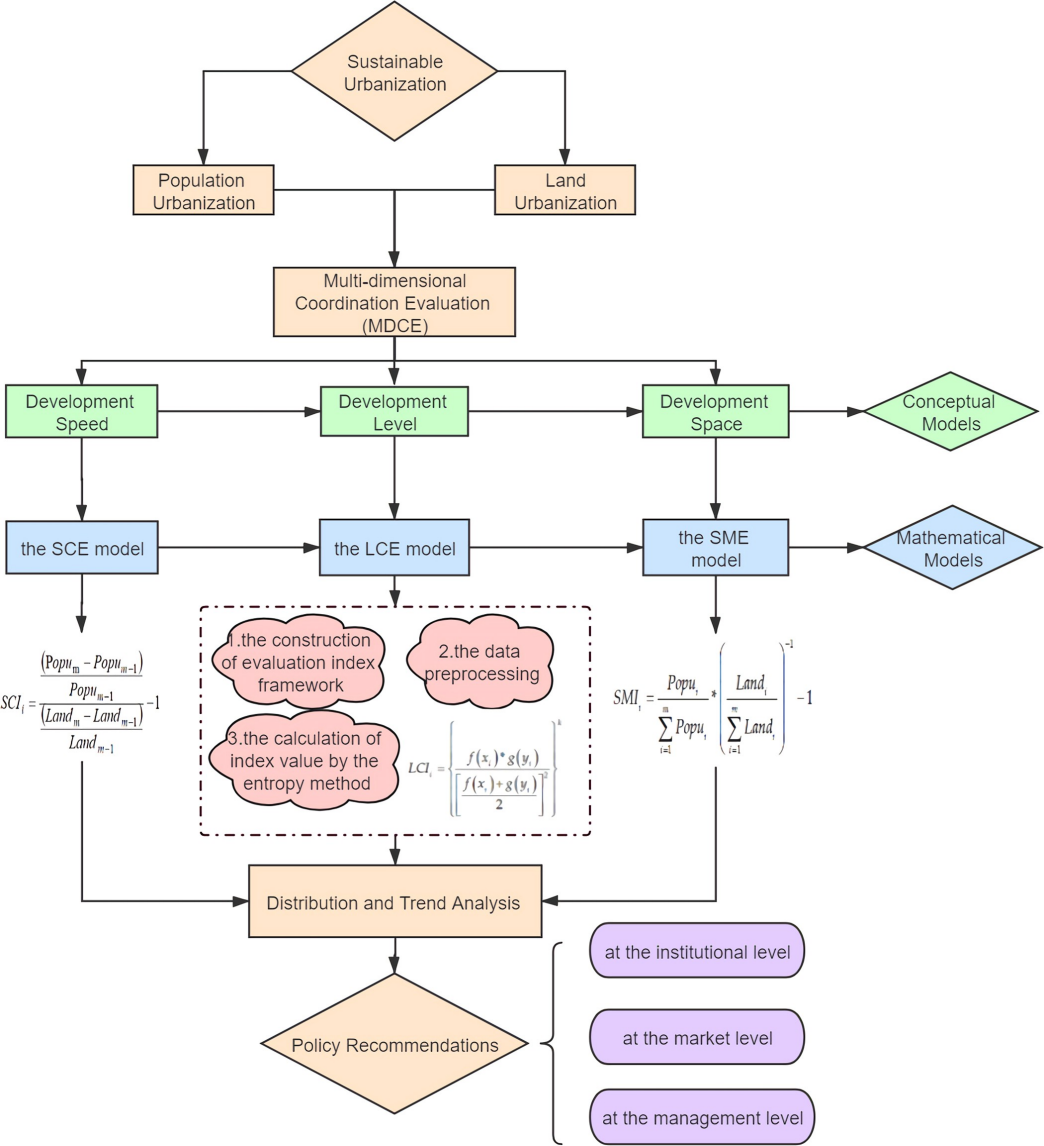
The framework of MDCE.

According to the above concept definitions and the related literatures [61], the MDCE model is constructed to analyze the coordination relationship between population urbanization and land urbanization (Fig 2). It is divided into three conceptual models and three mathematical models. In this paper, the multi-dimensional coordination relationship between population-land urbanization is evaluated from the speed-level-space perspective, so these three conceptual models involve three aspects of multi-dimensional coordination evaluation (i.e., the development speed, level and space), which could describe the coordination of population-land urbanization more comprehensively. Three mathematical models include the speed coordination evaluation (SCE) model, the level consistency evaluation (LCE) model and the space matching evaluation (SME) model. Furthermore, the LCE model contains 3 steps: the construction of evaluation index framework, the data preprocessing, and the calculation of index value by the entropy method. The three mathematical models play an equally important role in evaluating the coordinated development of population urbanization and land urbanization. The model is:

MDCEI=f(SCI,LCI,SMI) (1)

where *MDCE* is the multi-dimensional coordination evaluation index, *SCI* is the speed coordination index, *LCI* is the level consistency index and *SMI* is the space matching index.

#### 3.3.1. Speed coordination evaluation model

The speed coordination index *SCI*_*i*_ can be described by constructing the population-land urbanization speed coordination evaluation model:

$$SCI_{i}=[(Popu_{m}‐Popu_{m-1})/Popu_{m-1}]*{\left[(Land_{m}-Land_{m-1})/Land_{m-1}\right]}^{-1}-1$$ (2)

where *SCI*_*i*_ is the speed coordination index of the *i-*th region. *Popu*_*m*_ and *Land*_*m*_ are the number of urban population and the area of urban land in the *m*-th year of the region, respectively.

*SCI*_*i*_ is less than zero, indicating that the urban population growth rate in the *m*-th year is lower than the urban land growth rate. While *SCI*_*i*_ is greater than zero, this indicates that the urban population growth rate in the *m*-th year is greater than the urban land growth rate. *SCI*_*i*_ is equal to zero, indicating that the two are in a good coordination state.

#### 3.3.2. Level consistency evaluation model

*(1) Evaluation framework*. In terms of population urbanization, most scholars use the proportion of urban population to total population and population density to characterize population urbanization. Lin et al. believed that the connotation of population urbanization should include population capacity, quality, employment structure and quality of life, so they built the index system of population urbanization from three aspects of population composition, population quality and living standards [46]. However, the consumption expenditure and public service demand of urban residents are not considered in these studies. Therefore, this paper selects 7 indicators from five aspects of population composition (proportion of urban population to total population and proportion of employment in the secondary and tertiary industry), industrial structure (proportion of the added value of secondary and tertiary industry to GDP), income level (per capita annual disposable income of urban households), consumption level (per capita annual consumption expenditure of urban households) and public service (number of doctors per 10000 persons and number of college and university student per 10000 persons) to construct the evaluation index system of population urbanization.

In terms of land urbanization, most scholars prefer to use urban land scale and urban construction level to characterize land urbanization. Some literature also selected indicators such as the proportion of built-up areas to urban areas, per capita area of roads, per land total annual investment in fixed assets, per land general public budget expenditure and per land of the added value of secondary and tertiary industry [62], but ignored ecological environment and land output in the process of land urbanization index construction. Therefore, on the basis of relevant literature, this paper increases the coverage rate of urban green areas and per land general public budget revenue. Consequently, the index system of land urbanization is established from four parts: land structure (proportion of built-up areas to urban areas and coverage rate of urban green areas), land use scale (per capita area of roads), land investment (per land total annual investment in fixed assets and per land general public budget expenditure) and land output (per land general public budget revenue and per land of the added value of secondary and tertiary industry).

Eventually, on the basis of the above-mentioned comprehensive connotation of urbanization and combined with existing evaluation index system [61, 63], the population-land urbanization coordinated development evaluation framework is constructed by following the systematic and scientific principle. The evaluation framework is divided into three levels: goal, criterion and indicator. As shown in Table 1:

**Table 1:** Evaluation framework of the coordinated development of population-land urbanization.

Goal	criterion	indicator	unit	Literature sources
Population Urbanization	Population composition	Proportion of urban population to total population	%	[64]
		Proportion of employ-ment in the secondary and tertiary industry	%	[65]
	Industrial structure	Proportion of the added value of secondary and tertiary industry to GDP	%	[66]
	Income level	Per capita annual disposable income of urban households	yuan	[67]
	Consumption level	Per capita annual consumption expenditure of urban households	yuan	[68]
	Public service	Number of doctors per 10 000 persons	person	[69]
		Number of college and university student per 10000 persons	person	[70]
Land Urbanization	Land structure	Proportion of built-up areas to urban areas	%	[71]
		Coverage rate of urban green areas	%	[72]
	Land use scale	Per capita area of roads	m^2^	[73]
	Land investment	Per land total annual investment in fixed assets	yuan/m^2^	[74]
		Per land general public budget expenditure	yuan/m^2^	[75]
	Land output	Per land general public budget revenue	yuan/m^2^	[76]
		Per land of the added value of secondary and tertiary industry	yuan/m^2^	[77]

*(2) Data standardization*. In the comprehensive evaluation processes of the population urbanization and land urbanization, different evaluation indicators have different dimensions and orders of magnitude [35]. In this paper, data were preprocessed by standardizing the target value to eliminate the influence of different dimensions and orders of magnitude on the evaluation indicators. The standardization is as follows:

$$X_{i}^{'}=\{\begin{array}{l} {}^{\frac{X_{i}}{X_{0}}}\;(X_{i}<X_{0})\\ 1\;(X_{i}>X_{0}) \end{array}$$ (3)

Among them, $X_{i}^{\prime }$ indicates the standardized value of the *i*-th index, *X*_*i*_ indicates the actual value of the *i*-th index, and *X*_0_ indicates the target value of the *i*-th index.

*(3) Entropy method*. The entropy method is a weighting method based on the dispersion degree of the evaluation index data to objectively calculate the index weight [66]. It can be used to analyze the coordination relationship between population urbanization and land urbanization. Based on the information entropy and variations in the indicators, the weight of each indicator could be calculated. The specific calculation steps are as follows:

① *P*_*ij*_ is the proportion of the index value of the *i*-th indicator under the *j*-th indicator [77, 78]:
$$P_{ij}=X_{ij}^{'}*{\left(\sum_{i=1}^{m}X_{ij}^{i}\right)}^{-1}$$ (4)
② *e*_*j*_ denotes the information entropy of the *j*-th indicator, *d*_*j*_ denotes the entropy redundancy of the *j*-th indicator:
$$e_{j}=‐k\sum_{i=1}^{m}P_{ij}lnP_{ij}$$ (5)
$$d_{j}=1-e_{j}$$ (6)
  where $P_{ij}=X_{ij}^{'}*{\left(\sum_{i=1}^{m}X_{ij}^{i}\right)}^{-1}$, *k* = 1/*lnm*. When *lnP*_*ij*_ = 0, *e*_*j*_ = 0. The difference between 1 and the information entropy *e*_*j*_ is the entropy redundancy *d*_*j*_, which represents the size of the weight.
③ *a*_*j*_ is the weight of the *j*-th indicator:
$$a_{j}=d_{j}{\left(\sum_{i=1}^{m}d_{j}\right)}^{-1}$$ (7)
④ Using the entropy method to get the weight of each indicator. In general, the larger the difference coefficient is, the larger the index weight, and the greater the importance of the evaluation results.

*(4) Index of population urbanization and land urbanization*. *f*(*x*_*i*_) represents the population urbanization index and *g*(*y*_*i*_) represents the land urbanization index. *f*(*x*_*i*_) and *g*(*y*_*i*_) are defined as follows:

$$f(x_{i})=\sum_{i=1}^{m}a_{i}x_{{}_{i}}^{'}$$ (8)

$$g(y_{i})=\sum_{i=1}^{m}b_{i}y_{{}_{i}}^{'}$$ (9)

where *i* is the number of indicators, $x_{i}^{'}$ and $y_{i}^{'}$ are the standardized values of the population urbanization target value and land urbanization target value respectively. *a*_*i*_ is the weight of the population urbanization index, and *b*_*i*_ is the weight of the land urbanization index.

*(5) Calculation the level consistency index*. The level consistency index *LCI*_*i*_ is a quantitative indicator for measuring the coordinated development level of population urbanization and land urbanization, calculated as follows:

$$LCI_{i}={\left\{\frac{f(x_{i})*g(y_{i})}{{\left[\frac{f(x_{i})+g(y_{i})}{2}\right]}^{2}}\right\}}^{k}$$ (10)

where 0≤*LCI*_*i*_≤1, the closer the *LCI*_*i*_ is to 1, the greater the coordination development degree between the two systems of population urbanization and land urbanization. *k* is the adjusting coefficient, here *k* = 1/2.

#### 3.3.3. Space matching evaluation model

The space matching index *SMI*_*i*_ can be described by constructing the population urbanization and land urbanization space matching evaluation model:

$$SMI_{i}=\frac{Popu_{i}}{\sum_{i=1}^{m}Popu_{i}}*{\left(\frac{Land_{i}}{\sum_{i=1}^{m}Land_{i}}\right)}^{-1}-1$$ (11)

where *SMI*_*i*_ is the space matching index of the *i*-th region, *Popu*_*i*_ and *Land*_*i*_ are the spatial distribution values of the urban population and the urban land area in the *i*-th region, respectively.

Furthermore, if it is a whole region, the comprehensive space matching evaluation model is used to define this region:

$$ZSMI_{i}={\left(\frac{\sum_{i=1}^{m}{\left(SMI_{i}\right)}^{2}}{m}\right)}^{\frac{1}{2}}$$ (12)

where *ZSMI*_*i*_ is the population-land urbanization space matching index in the superior region, *SMI*_*i*_ is the population-land urbanization space matching index in the lower region, and *m* is the number of the lower region.

#### 3.3.4. Types of MDCE indicators

As shown in the Table 2, on the basis of the uniform distribution method, the speed coordination evaluation indicators are divided into five categories (i.e., high, medium-high, medium, medium-low and low coordination). Referred to previous evaluation criteria [79], the level consistency evaluation indicators are classified into three types of ten intervals to achieve coordinated development. And according to the principles of basic equalization and approximate rounding, the space matching evaluation indicators are divided into five categories (i.e., high, medium-high, medium, medium-low and low matching).

**Table 2:** Classification of population-land urbanization SCE, LCE and SME indicators.

Indicator	Classification	Type	
***SCI***_*i*_	0≤*Abs*(*SCI*_*i*_)<0.2	High Coordination	
	0.2≤*Abs*(*SCI*_*i*_)<0.4	Medium-High Coordination	
	0.4≤*Abs*(*SCI*_*i*_)<0.6	Medium Coordination	
	0.6≤*Abs*(*SCI*_*i*_)<0.8	Medium-Low Coordination	
	*Abs*(*SCI*_*i*_)≥0.8	Low Coordination	
***LCI***_*i*_	0≤*LCI*_*i*_<0.1	Extremely Imbalanced	Imbalanced recession interval
	0.1≤*LCI*_*i*_<0.2	Moderately Imbalanced	
	0.2≤*LCI*_*i*_<0.3	Slightly Imbalanced	
	0.3≤*LCI*_*i*_<0.4	Barely Harmonic Coordination	Transitional harmonic interval
	0.4≤*LCI*_*i*_<0.5	Harmonic Coordination	
	0.5≤*LCI*_*i*_<0.6	Primary Coordination	Coordinated development interval
	0.6≤*LCI*_*i*_<0.7	Intermediate Coordination	
	0.7≤*LCI*_*i*_<0.8	Good Coordination	
	0.8≤*LCI*_*i*_<0.9	High Quality Coordination	
	0.9≤*LCI*_*i*_<1	Extremely Coordination	
***SMI***_*i*_	0≤*Abs*(*SMI*_*i*_)<0.1	High Matching	
	0.1≤*Abs*(*SMI*_*i*_)<0.3	Medium-High Matching	
	0.3≤*Abs*(*SMI*_*i*_)<0.6	Medium Matching	
	0.6≤*Abs*(*SMI*_*i*_)<0.9	Medium-Low Matching	
	*Abs*(*SMI*_*i*_)≥0.9	Low Matching	

## 4. Results

### 4.1 MDCE based on the temporal pattern

The multi-dimensional coordinated development levels of the population-land urbanization in the YEB from 2007–2017 were evaluated in this section. Specifically, the section studied the speed coordination level, level consistency degree and space matching degree of the region.

As shown in Table 3, the speed coordination level in the YEB had significantly fluctuated during these 11 years. Most types of *SCI*_*i*_ were medium-high and medium coordinations. The *SCI*_*i*_ had been upgraded from -0.3054 in 2007 to -0.1961 in 2017, indicating that the speed coordination level of population-land urbanization in the YEB had been gradually optimized. In addition, the *SCI*_*i*_ was less than 0, meaning that during this period the urban population growth rate was lower than the urban land growth rate and the development of land urbanization was too fast. The level consistency degree in the YEB was at a relatively high level, and the type of *LCI*_*i*_ was high-quality coordination. The *LCI*_*i*_ had been shifted from 0.9789 in 2007 to 0.9752 in 2017, demonstrating that the development level of population-land urbanization in this area was indeed stable and at a high level. Also, the population urbanization development index was lower than the land urbanization development index, revealing that the population urbanization was lagged behind. Types of *SMI*_*i*_ in the YEB were mostly high matching in these 11 years, which showed that the space matching degree of population-land urbanization in this area was pretty high. The *SMI*_*i*_ increased from 0.0870 in 2007 to 0.0822 in 2017, displaying that the population-land urbanization space matching degree was on the rise. Additionally, the *SMI*_*i*_ was greater than 0, indicating that the spatial distribution ratio of urban population was greater than that of urban land (i.e., urban population was concentratedly distributed).

**Table 3:** MDCE of population-land urbanization in the YEB from 2007 to 2017.

Year	*f(x)*	*g(y)*	*SCI*_*i*_	*LCI*_*i*_	*SMI*_*i*_
**2007**	0.3359	0.5087	-0.3054	0.9789	0.0870
**2008**	0.3514	0.5383	-0.4189	0.9777	0.1032
**2009**	0.3765	0.5935	-0.2947	0.9747	0.0970
**2010**	0.3950	0.6205	-0.2843	0.9750	0.0788
**2011**	0.4199	0.6394	-0.5761	0.9783	0.0595
**2012**	0.4498	0.6815	-0.2984	0.9788	0.0696
**2013**	0.4798	0.7309	-0.3937	0.9782	0.0850
**2014**	0.5043	0.7905	-0.6121	0.9753	0.0609
**2015**	0.5369	0.8667	-0.2529	0.9720	0.0776
**2016**	0.5647	0.9183	-0.3676	0.9712	0.0814
**2017**	0.5927	0.9294	-0.1961	0.9752	0.0822

### 4.2 MDCE based on the spatial pattern

From the provincial perspective, the multi-dimensional coordination of population-land urbanization had been evaluated in terms of the speed coordination, level consistency and space matching in the YEB. In order to better analyze them, we selected 2007, 2010, 2013, 2017 as representatives for in-depth research.

#### 4.2.1 Speed coordination evaluation

Shanghai was excluded from the analysis of the speed coordination evaluation due to the small change in the built-up area from 2007 to 2017 (886km^2^, 886 km^2^, 886 km^2^, 998.8 km^2^, 998.8 km^2^, 998.8 km^2^, 998.8 km^2^, 998.8 km^2^, 998.8 km^2^, 998.8 km^2^ and 998.8 km^2^).

**Fig 3:**
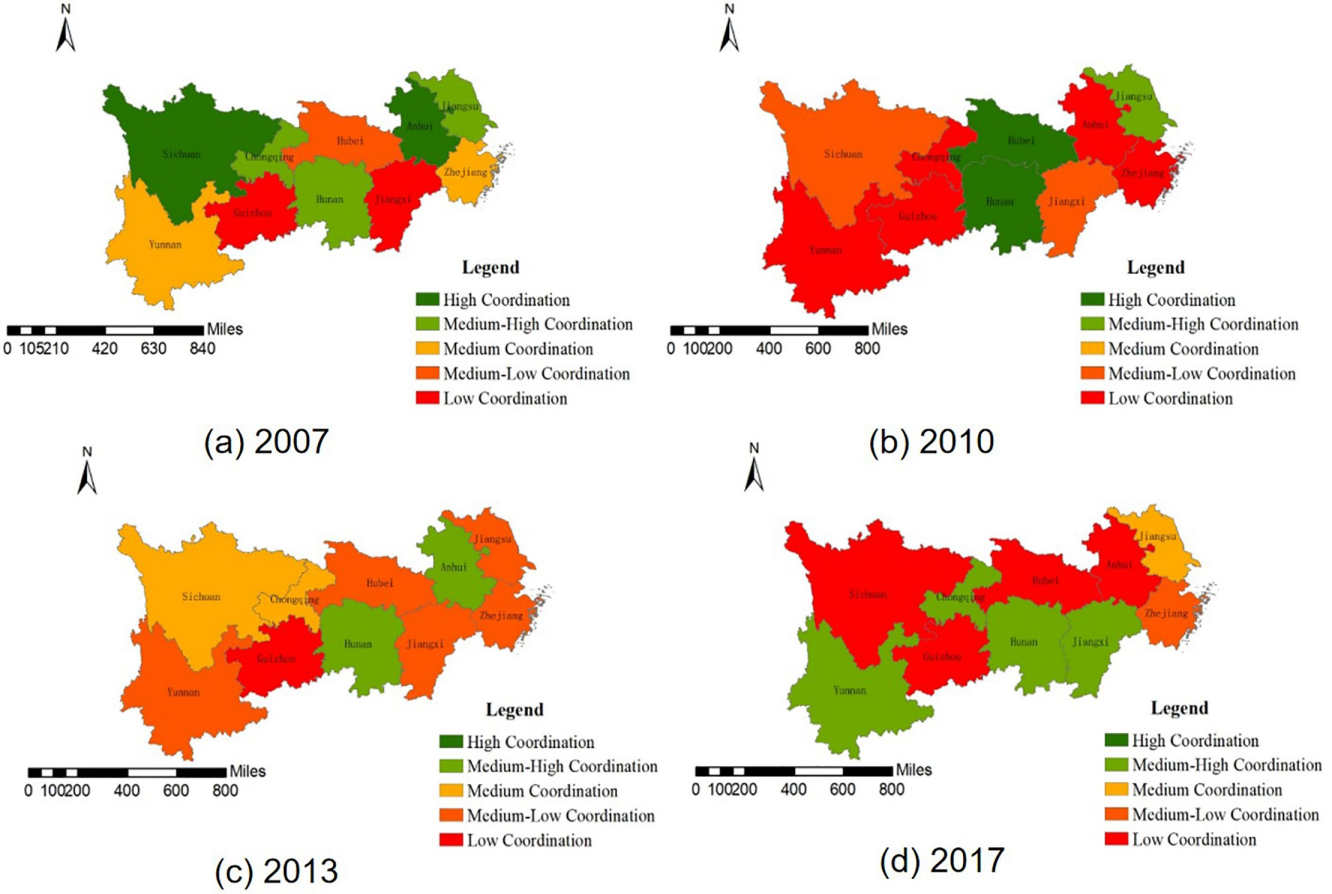
Speed coordination evaluation of the population-land urbanization in the YEB in 2007, 2010, 2013, 2017. a. 2007. b.2010. c.2013. d.2017. (Quoted from the Tianditu map. Source of base map: the open source map data service provided by the National Platform for Common GeoSpatial Information Services (https://www.tianditu.gov.cn).

In Table 4 and Fig 3, in general, from 2007 to 2017, the speed coordinated development of population-land urbanization in the provinces of the YEB had decreased overall. Specifically, the speed coordination of Jiangsu, Zhejiang, Anhui, Hubei, Chongqing, Sichuan and Guizhou was reduced, and the speed coordination of Jiangxi, Hunan and Yunnan was improved, and Sichuan had the largest change.

**Table 4:** Speed coordination evaluation of the population-land urbanization in the YEB in 2007, 2010, 2013, 2017.

	2007				2010			
**Province**	**UPGR**	**ULGR**	***SCI***_***i***_	**TSC**	**UPGR**	**ULGR**	***SCI***_***i***_	**TSC**
**Jiangsu**	0.0341	0.0527	-0.3528	MHC	0.0979	0.0786	0.2452	MHC
**Zhejiang**	0.0290	0.0559	-0.4817	MC	0.0985	0.0468	1.1039	LC
**Anhui**	0.0446	0.0382	0.1660	HC	-0.0074	0.0713	-1.1032	LC
**Jiangxi**	0.0359	0.0127	1.8258	LC	0.0273	0.1040	-0.7375	MLC
**Hubei**	0.0125	0.0346	-0.6385	MLC	0.0820	0.0916	-0.1044	HC
**Hunan**	0.0473	0.0754	-0.3733	MHC	0.0282	0.0304	-0.0734	HC
**Chongqing**	0.0382	0.0571	-0.3309	MHC	0.0370	0.2288	-0.8381	LC
**Sichuan**	0.0325	0.0381	-0.1469	HC	0.0202	0.0814	-0.7519	MLC
**Guizhou**	0.0122	0.1635	-0.9251	LC	0.1126	-0.1642	-1.6858	LC
**Yunnan**	0.0432	0.0755	-0.4273	MC	0.0307	0.0064	3.7676	LC
	**2013**				**2017**			
**Province**	**UPGR**	**ULGR**	***SCI***_***i***_	**TSC**	**UPGR**	**ULGR**	***SCI***_***i***_	**TSC**
**Jiangsu**	0.0200	0.0547	-0.6340	MLC	0.0193	0.0324	-0.4058	MC
**Zhejiang**	0.0168	0.0720	-0.7673	MLC	0.0272	0.0761	-0.6422	MLC
**Anhui**	0.0366	0.0551	-0.3353	MHC	0.0388	0.0186	1.0819	LC
**Jiangxi**	0.0328	0.0963	-0.6597	MLC	0.0349	0.0519	-0.3278	MHC
**Hubei**	0.0224	0.0926	-0.7581	MLC	0.0236	0.1315	-0.8205	LC
**Hunan**	0.0362	0.0301	0.2012	MHC	0.0411	0.0325	0.2666	MHC
**Chongqing**	0.0326	0.0599	-0.4562	MC	0.0326	0.0533	-0.3882	MHC
**Sichuan**	0.0353	0.0743	-0.5254	MC	0.0371	0.0080	3.6318	LC
**Guizhou**	0.0444	-0.4609	-1.0964	LC	0.0497	-0.0914	-1.5439	LC
**Yunnan**	0.0358	0.1757	-0.7961	MLC	0.0434	0.0313	0.3844	MHC

**Abbreviations**: **UPGR** represents urban population growth rate, **ULGR** urban land growth rate, **TSC** type of speed coordination, **LC** low coordination, **MLC** medium-low coordination, **MC** medium coordination, **MHC** medium-high coordination, and **HC** high coordination.

Among 10 provinces in the YEB, the urban land growth rate of most provinces in 2007 was faster than that of the urban population. Only *SCI*_*i*_ of Anhui and Jiangxi was greater than 0, indicating that urban population growth rate was faster than urban land growth rate. The speed coordination type of Anhui and Sichuan was high coordination, while that of Jiangsu, Hunan and Chongqing was medium-high coordination. The coordinations of Zhejiang and Yunnan were medium, Hubei’s medium-low, and Jiangxi and Guizhou’s low coordinations. Moreover, Sichuan had the highest speed coordination level of population-land urbanization with a value of -0.1469, and Jiangxi had the lowest speed coordination level of population-land urbanization with a value of 1.8258.

The growth rate of urban land in most provinces of the YEB was faster than that of urban population in 2010. Only *SCI*_*i*_ of Jiangsu, Zhejiang and Yunnan was greater than 0, showing that urban population growth rate was faster than urban land growth rate. The speed coordination type of Hubei and Hunan was high, Jiangsu’s medium-high, Jiangxi and Sichuan’s medium-low, and Zhejiang, Anhui, Chongqing, Guizhou and Yunnan’s low. Furthermore, Hunan had the highest speed coordination level of population-land urbanization with a value of -0.0734, and Yunnan had the lowest speed coordination level of population-land urbanization with a value of 3.7676.

In Table 4, the growth rate of urban land in most provinces of the YEB was faster than that of urban population in 2013. Only the *SCI*_*i*_ of Hunan was above 0, displaying that urban population growth rate was faster than urban land growth rate. The speed coordination type of Anhui and Hunan was medium-high coordination, Chongqing and Sichuan’s medium, Jiangsu, Zhejiang, Jiangxi, Hubei and Yunnan’s medium-low, and Guizhou’s low coordination. Further, Hunan had the highest speed coordination level of population-land urbanization with a value of 0.2012, while Guizhou had the lowest speed coordination level of population-land urbanization with a value of -1.0964.

The growth rate of urban land in Jiangsu, Zhejiang, Jiangxi, Hubei, Chongqing and Guizhou was faster than that of urban population in 2017. The *SCI*_*i*_ of Anhui, Hunan, Sichuan and Yunnan was more than 0, urban population growth rate was faster than urban land growth rate. From the perspective of population-land urbanization speed coordination type, Jiangxi, Hunan, Chongqing and Yunnan were medium-high coordination, Jiangsu’s was medium coordination, Zhejiang’s was medium-low coordination and Anhui, Hubei, Sichuan and Guizhou’s were low coordination. Besides, Hunan had the highest speed coordination level of population-land urbanization with a value of 0.2666, Sichuan had the lowest speed coordination level of population-land urbanization with a value of 3.6318.

#### 4.2.2 Level consistency evaluation

**Fig 4:**
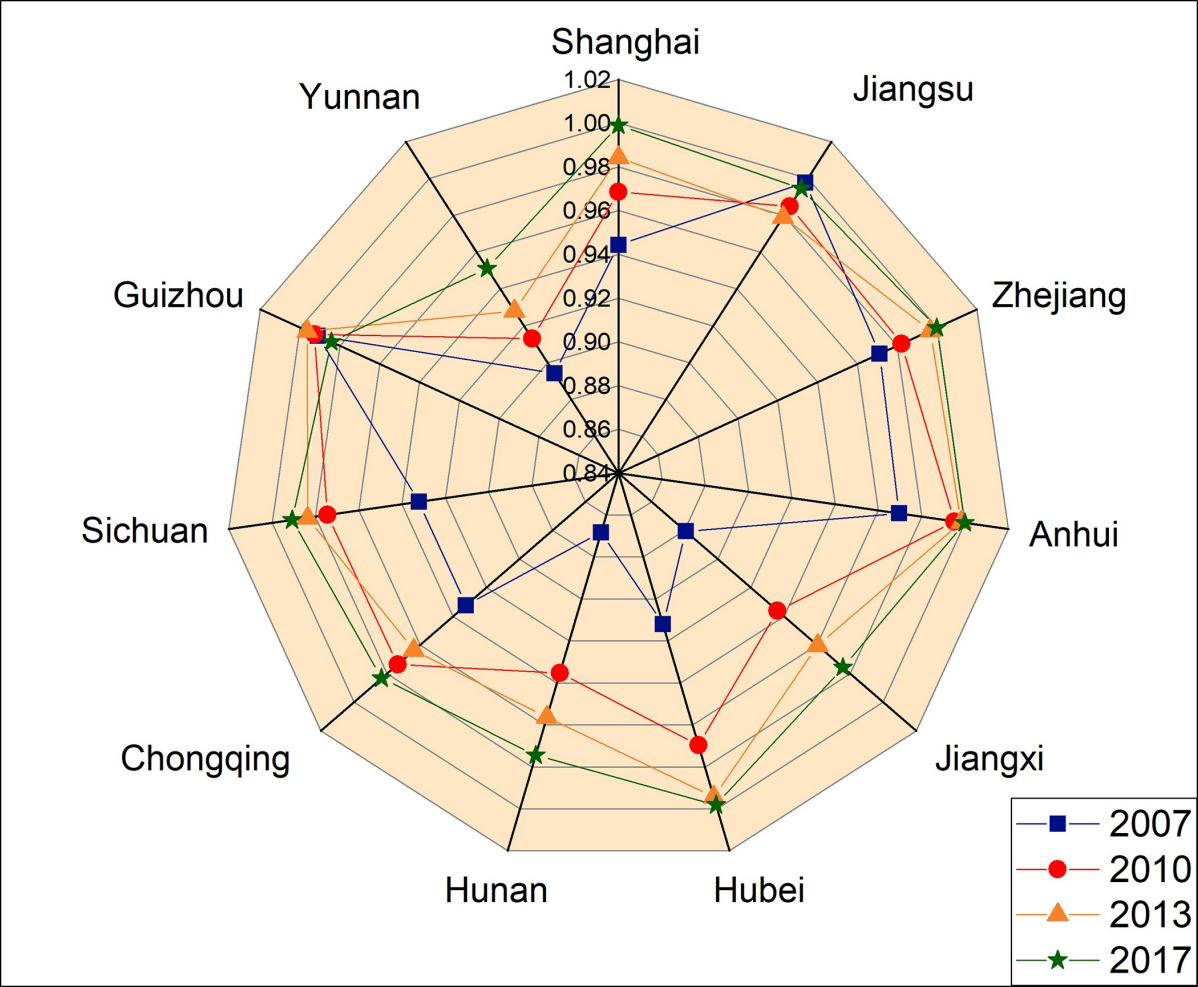
Level consistency evaluation of the population-land urbanization in the YEB in 2007, 2010, 2013, 2017.

It can be seen from Table 5 and Fig 4 that the population urbanization index of Shanghai, Jiangsu and Guizhou was smaller than the land urbanization index in 2007 and 2010, while the population urbanization indices of Shanghai and Jiangsu were smaller than the land urbanization indices in 2013 and 2017. The population-land urbanization level consistency degree, the population urbanization index, and the land urbanization index of these provinces all showed an upward trend, demonstrating that the socio-economic development of the YEB was growing rapidly, the urban population was constantly gathering, and the urban land was constantly expanding.

**Table 5:** Level consistency evaluation of the population-land urbanization in the YEB in 2007, 2010, 2013, 2017.

	*2007*			*2010*		
Province	*f(x)*	*g(y)*	*LCI*_*i*_	*f(x)*	*g(y)*	*LCI*_*i*_
**Shanghai**	0.4887	0.9681	0.9443	0.5852	0.9722	0.9686
**Jiangsu**	0.3734	0.4248	0.9979	0.4352	0.6171	0.9849
**Zhejiang**	0.4460	0.2741	0.9711	0.5242	0.3583	0.9822
**Anhui**	0.2865	0.1738	0.9696	0.3480	0.2861	0.9952
**Jiangxi**	0.2743	0.0979	0.8806	0.3254	0.1561	0.9361
**Hubei**	0.3178	0.1330	0.9120	0.3709	0.2253	0.9697
**Hunan**	0.3210	0.1081	0.8683	0.3695	0.1763	0.9352
**Chongqing**	0.3712	0.1740	0.9323	0.4455	0.2799	0.9736
**Sichuan**	0.2791	0.1307	0.9322	0.3448	0.2185	0.9745
**Guizhou**	0.2523	0.3300	0.9911	0.3065	0.3905	0.9927
**Yunnan**	0.2517	0.0960	0.8941	0.3274	0.1377	0.9130
	***2013***			***2017***		
**Province**	***f(x)***	***g(y)***	***LCI***_***i***_	***f(x)***	***g(y)***	***LCI***_***i***_
**Shanghai**	0.6820	0.9744	0.9843	0.8913	0.9768	0.9990
**Jiangsu**	0.5454	0.8243	0.9791	0.6679	0.8229	0.9946
**Zhejiang**	0.6299	0.5355	0.9967	0.7697	0.7525	0.9999
**Anhui**	0.4255	0.3776	0.9982	0.5199	0.5186	0.9999
**Jiangxi**	0.3877	0.2188	0.9604	0.4820	0.3088	0.9757
**Hubei**	0.4420	0.3565	0.9943	0.5456	0.4828	0.9981
**Hunan**	0.4911	0.2688	0.9563	0.6113	0.3870	0.9744
**Chongqing**	0.6482	0.3753	0.9638	0.7258	0.5024	0.9833
**Sichuan**	0.4177	0.2899	0.9836	0.5052	0.3842	0.9907
**Guizhou**	0.3703	0.3122	0.9964	0.5211	0.3647	0.9843
**Yunnan**	0.4159	0.1902	0.9281	0.5255	0.2774	0.9511

For more detailed analysis, the YEB was divided into three regions (i.e., the upper, middle and lower region). The upper region included Chongqing, Sichuan, Guizhou and Yunnan, the middle region Anhui, Jiangxi, Hubei and Hunan, and the lower region Shanghai, Zhejiang and Jiangsu.

For the development of the YEB’s upper region in these four years, Chongqing’s *LCI*_*i*_ increased from 0.9323 in 2007 to 0.9833 in 2017, Sichuan’s *LCI*_*i*_ increased from 0.9322 in 2007 to 0.9907 in 2017, Guizhou’s *LCI*_*i*_ increased from 0.9911 in 2007 to 0.9843 in 2017, and the level consistency type of Chongqing, Sichuan and Guizhou was extremely coordination. Yunnan’s *LCI*_*i*_ increased from 0.8941 in 2007 to 0.9511 in 2017, and its level consistency type changed from high-quality coordination to extremely coordination. In general, the population urbanization index and the land urbanization index in the upper region were relatively low, but the coordinated development level of population-land urbanization was indeed high.

For the development of the YEB’s middle region in these four years, Anhui ’s *LCI*_*i*_ increased from 0.9696 in 2007 to 0.9999 in 2017, indicating that the population-land urbanization coordination level in Anhui was relatively high, and the type was extremely coordination. Jiangxi’s *LCI*_*i*_ rose from 0.8806 in 2007 to 0.9757 in 2017 and its population urbanization index and land urbanization index showed an increasing trend. But both were at low levels. Hubei’s *LCI*_*i*_ increased from 0.9120 in 2007 to 0.9981 in 2017, and Hunan’s *LCI*_*i*_ rose from 0.9563 in 2007 to 0.9744 in 2017. The index of population-land urbanization in Hunan showed an upward trend, but it was at a low level. In general, the population urbanization index and the land urbanization index in the middle region were both low, but the growth rates of these two indices were relatively fast.

For the development of the YEB’s lower region in these four years, Shanghai had the highest population urbanization index and the highest land urbanization index and its *LCI*_*i*_ rose from 0.9443 in 2007 to 0.9990 in 2017. Jiangsu’s *LCI*_*i*_ decreased from 0.9979 in 2007 to 0.9946 in 2017. The change was comparatively flat. Zhejiang’s *LCI*_*i*_ increased from 0.9711 in 2007 to 0.9999 in 2017, its population urbanization index increased from 0.4460 to 0.7697, and the land urbanization index increased from 0.2741 to 0.7525. Generally speaking, the population urbanization index and the land urbanization index of the lower region were both high, and the coordinated development level of population-land urbanization was also high.

#### 4.2.3 Space matching evaluation

As shown in Table 6, the space matching type of population-land urbanization in Zhejiang, Hubei and Sichuan in 2007 was high matching. Shanghai, Jiangsu, Anhui, Jiangxi, Hunan, and Chongqing’s space matchings were medium-high and the ones of Guizhou and Yunnan were medium. Further, Hubei had the highest space matching degree of urban population and urban land with a value of -0.0387. However, Yunnan had the lowest space matching degree with a value of 0.4646. The *SMI*_*i*_ of Shanghai, Jiangsu, Zhejiang, Anhui, Hubei and Chongqing were less than 0 (i.e., the proportion of urban population spatial distribution was smaller than the proportion of urban land spatial distribution), indicating that these areas were urban land concentrated.

**Table 6:** Space matching evaluation of the population-land urbanization in the YEB in 2007, 2010, 2013, 2017.

	2007				2010			
Province	UPP	ULP	*SMI*_*i*_	TSM	UPP	ULP	*SMI*_*i*_	TSM
**Shanghai**	0.0738	0.0872	-0.1538	MHM	0.0736	0.0824	-0.1074	MHM
**Jiangsu**	0.1657	0.1869	-0.1136	MHM	0.1706	0.1934	-0.1179	MHM
**Zhejiang**	0.1189	0.1246	-0.0457	HM	0.1201	0.1198	0.0026	HM
**Anhui**	0.0955	0.1097	-0.1291	MHM	0.0917	0.1152	-0.2039	MHM
**Jiangxi**	0.0701	0.0549	0.2765	MHM	0.0704	0.0613	0.1486	MHM
**Hubei**	0.1018	0.1059	-0.0387	HM	0.1019	0.0983	0.0368	HM
**Hunan**	0.1037	0.0856	0.2107	MHM	0.1018	0.0866	0.1761	MHM
**Chongqing**	0.0549	0.0657	-0.1638	MHM	0.0547	0.0717	-0.2368	MHM
**Sichuan**	0.1167	0.1101	0.0602	HM	0.1157	0.1095	0.0564	HM
**Guizhou**	0.0414	0.0301	0.3733	MM	0.0421	0.0231	0.8235	MLM
**Yunnan**	0.0575	0.0393	0.4646	MM	0.0573	0.0387	0.4825	MM
	**2013**				**2017**			
**Province**	**UPP**	**ULP**	***SMI***_***i***_	**TSM**	**UPP**	**ULP**	***SMI***_***i***_	**TSM**
**Shanghai**	0.0702	0.0690	0.0168	HM	0.0612	0.0568	0.0757	HM
**Jiangsu**	0.1651	0.2023	-0.1839	MHM	0.1592	0.1957	-0.1864	MHM
**Zhejiang**	0.1141	0.1172	-0.0259	HM	0.1109	0.1182	-0.0612	HM
**Anhui**	0.0936	0.1110	-0.1569	MHM	0.0965	0.1056	-0.0868	HM
**Jiangxi**	0.0717	0.0621	0.1548	MHM	0.0728	0.0645	0.1285	MHM
**Hubei**	0.1025	0.0978	0.0487	HM	0.1009	0.0954	0.0577	HM
**Hunan**	0.1041	0.0874	0.1908	MHM	0.1080	0.0814	0.3278	MM
**Chongqing**	0.0562	0.0770	-0.2700	MHM	0.0568	0.0809	-0.2977	MHM
**Sichuan**	0.1181	0.1158	0.0197	HM	0.1216	0.1287	-0.0551	HM
**Guizhou**	0.0430	0.0171	1.5097	LM	0.0475	0.0317	0.4999	MM
**Yunnan**	0.0615	0.0434	0.4169	MM	0.0646	0.0412	0.5699	MM

**Abbreviations**: **UPP** represents urban population proportion, **ULP** urban land proportion, **TSM** type of space matching, **LM** low matching, **MLM** medium-low matching, **MM** medium matching, **MHM** medium-high matching, and **HM** high matching.

The space matching type of population-land urbanization in Zhejiang, Hubei and Sichuan in 2010 was high matching. Shanghai, Jiangsu, Anhui, Jiangxi, Hunan, and Chongqing’s were medium-high matching, Yunnan’s medium matching, and Guizhou’s low matching. In addition, Zhejiang had the highest space matching degree of urban population and urban land with a value of 0.0026. Guizhou had the lowest space matching degree of urban population and urban land with a value of 0.8235. Compared with the space matching degree of the YEB in 2007, it had overall improved in 2010. The *SMI*_*i*_ of Zhejiang, Jiangxi, Hubei, Hunan, Sichuan, Guizhou and Yunnan were above 0, indicating that these areas were urban population concentrated.

In Table 6, the space matching type of population-land urbanization in Shanghai, Zhejiang, Hubei and Sichuan in 2013 was high matching. Jiangsu, Anhui, Jiangxi, Hunan, and Chongqing’s were medium-high matching, Yunnan’s medium matching and Guizhou’s low matching. Moreover, Shanghai had the highest space matching degree of urban population and urban land with a value of 0.0168, Guizhou had the lowest space matching degree with a value of 1.5097. The *SMI*_*i*_ of Jiangsu, Zhejiang, Anhui and Chongqing was less than 0, the spatial distribution of urban population was smaller than the proportion of urban land space distribution, indicating that these areas were urban land concentrated.

In 2017, Shanghai, Zhejiang, Anhui, Hubei and Sichuan’s space matching types were high matching, Jiangsu, Jiangxi, and Chongqing’s medium-high matching and Hunan, Guizhou and Yunnan’s medium matching. In addition, Sichuan had the highest space matching degree of urban population and urban land with a value of -0.0551. Yunnan had the lowest space matching degree of urban population and urban land with a value of 0.5699. The *SMI*_*i*_ of Shanghai, Jiangxi, Hubei, Hunan, Guizhou and Yunnan was above 0, and these were urban population concentrated.

**Fig 5:**
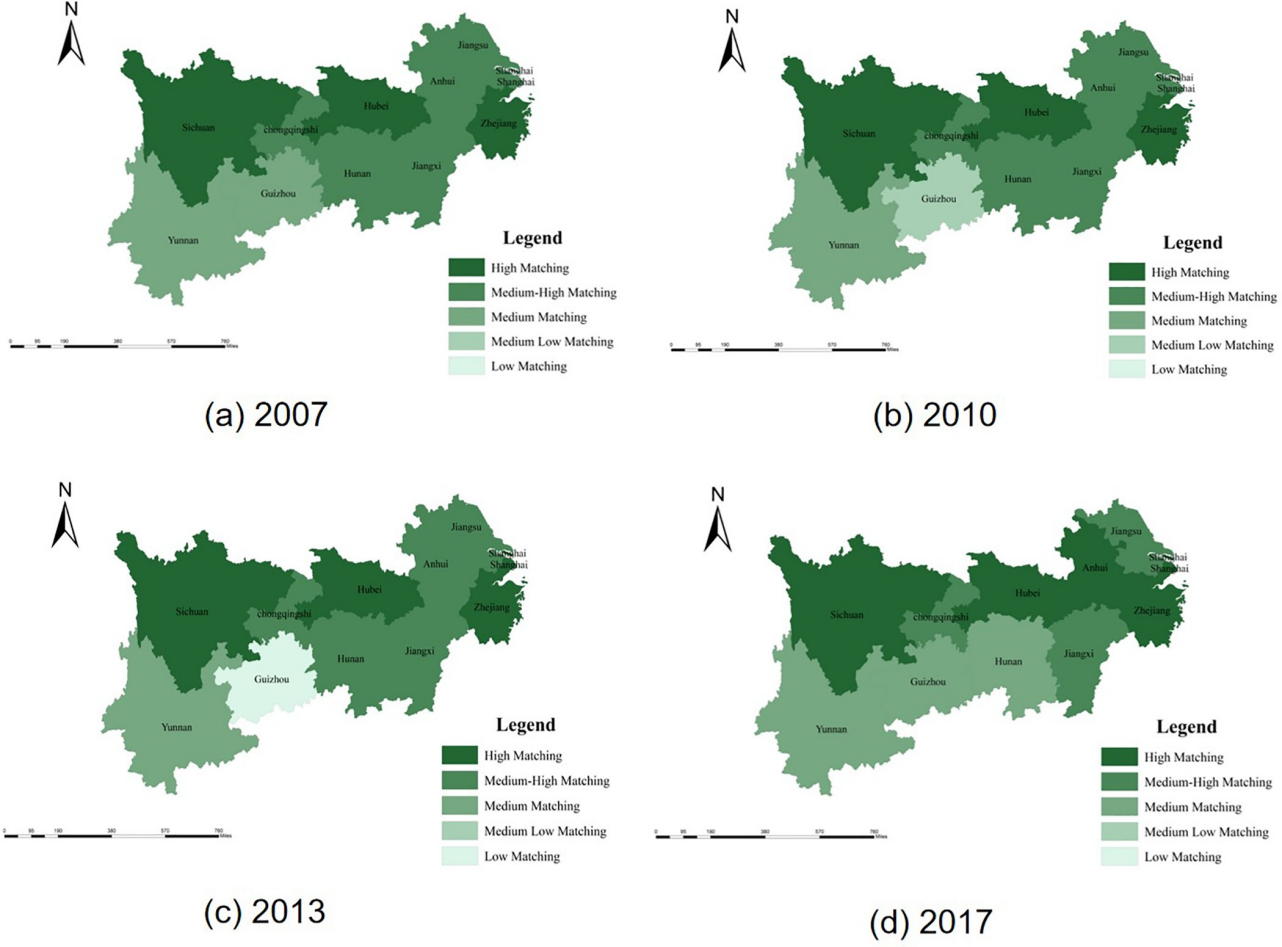
Space matching evaluation of the population-land urbanization in the YEB in 2007 to 2017. a. 2007. b.2010. c.2013. d.2017. (Quoted from the Tianditu map. Source of base map: the open source map data service provided by the National Platform for Common GeoSpatial Information Services (https://www.tianditu.gov.cn).

In order to show the spatial and temporal pattern of the space matching degree of the YEB in 2007, 2010, 2013 and 2017 more intuitively, ArcGIS 10.2 software was used to display the space matching results graphically. It can be seen from Fig 5 that the population-land urbanization space matching degree was high in the YEB, and the overall pattern was "Northeast-Southwest", the space matching degree was high in the northeast and low in the southwest.

## 5. Discussion and conclusion

Existing researches on population urbanization and land urbanization are becoming more and more abundant. Most scholars believe that the degree of coordination between population urbanization and land urbanization is gradually increasing, and the level of population urbanization lags behind the level of land urbanization [80–82], which is similar to our research. However, previous studies only discussed the single dimension of population-land urbanization, such as the level or speed of development, but ignored the spatial distribution of its development. Therefore, this paper constructs the MDCE model to explore the coordinated relationship between population and land urbanization from the multi-dimensional perspective of speed-level-space.

Highlights of this study are: i) establishing the MDCE model and using the entropy method to study the coordination relationship between population urbanization and land urbanization; ii) constructing an evaluation framework for coordinated development of population-land urbanization from the speed-level-space perspective; iii) using distribution and trend analysis mapping to make the spatial coordination more concise and intuitive; iv) identifying 11 provinces in the YEB during 10 years into 5 types; v) formulating joint management strategies at the institutional, market and management levels, according to the heterogeneity of regional development.

This paper established the MDCE model to evaluate the coordination relationship between population urbanization and land urbanization from the speed-level-space perspective. Specifically, the paper constructed the speed coordination model to research the development speed of population-land urbanization, built the level consistency model, applied the entropy method to research the development level of population-land urbanization, and used the space matching model to research the spatial distribution of population-land urbanization. The purpose of this study is to evaluate the coordination degree between population urbanization and land urbanization in the evolution of the spatial-temporal pattern, and to explore the development path to achieve sustainable urbanization for more regions.

From the perspective of time, the research results of this paper are: (1) the development of population urbanization and land urbanization from 2007 to 2017 in the YEB became more and more coordinated, and the development speed of population urbanization lagged behind that of land urbanization. This is mainly because the household registration system restricts the development of population urbanization, and the acceleration of the industrialization process has led to a large scale expansion of urban construction land and promoted the development of land urbanization. (2) the level consistency of population urbanization and land urbanization from 2007 to 2017 in the YEB were all high, but the development level of population urbanization was lower than that of land urbanization. This is mainly because with the development of the economy and society, the level of population urbanization and land urbanization have increased to a high level. In addition, the rapid development of real estate industry and land finance (i.e. the local government could obtain high profits from land transfer) promote the development of land urbanization to a higher level. (3) the space matching of population urbanization and land urbanization from 2007 to 2017 in the YEB had a high degree of coordination, and the space matching degree of population urbanization was higher than that of land urbanization. This is mainly because the process of urbanization has caused a large number of rural population moving into cities, leading to traffic congestion and housing shortages. Therefore, the area is densely populated and the land use efficiency needs to be further improved.

From the perspective of spatiality, the research results of this paper are: (1) the speed coordination between population urbanization and land urbanization in these 10 provinces had decreased overall from 2007 to 2017, and most provinces were characterized by excessive land urbanization development. The speed coordination of Jiangxi, Hunan and Yunnan was optimized, and Anhui, Hunan, Sichuan and Yunnan was over-developed in population urbanization. The reason is that driven by economic benefits, the development of land urbanization in most provinces is too fast. However, due to the complex terrain condition and a large number of rural population being attracted to cities, the development of population urbanization in Anhui, Hunan, Sichuan, Yunnan and other places is too fast. (2) the development level of population urbanization and land urbanization in 11 provinces from 2007 to 2017 remained stable and high, and the development level of population urbanization and land urbanization were continuously improved. Because of the sustained and healthy development of economy and society, the massive development of urban construction land, the rapid increase of population, and the continuous improvement of urban public services and infrastructure, the development level of population urbanization and land urbanization has been constantly improved. (3) the space matching of population urbanization and land urbanization had a decline in more than half of the 11 provinces from 2007 to 2017, but the change was small. There were 6 provinces (i.e., Shanghai, Jiangxi, Hubei, Hunan, Guizhou, Yunnan) were population-concentrated type and 5 provinces (i.e., Jiangsu, Zhejiang, Anhui, Chongqing, Sichuan) land-concentrated type.

## 6. Policy implications

Based the above research discussion and conclusion, the policy recommendations of this paper mainly focus on the following three aspects:

At the institutional level, the government should further deepen the reform of the household registration system, and establish an orderly rural population transfer mechanism. The household registration system is an important obstructive factor to the development of population urbanization. Therefore, it is urgent to eliminate the barriers to population urbanization in order to promote the coordinated development of population-land urbanization. The results showed that the development speed of the population urbanization lagged behind the land urbanization. Thus, the institutional barriers to urban population transfer should be broken, the population urbanization level should be improved, and a reasonable population urbanization mechanism should be established. At the same time, we need to vigorously improve the central city energy of the whole region, strengthen the industrial support between regions, and enhance the regional service function and the ability to absorb the population.

At the market level, a unified urban and rural land market should be constructed to enhance the intensive use of urban land. According to the results, the land urbanization had developed too fast. Therefore, the government should reasonably control the scale of cities, establish a unified urban and rural land market in the region, and control the disorderly expansion of urban land. At the same time, the urban population introduction mechanism should be further innovated to ensure the coordinated and sustainable development of population urbanization and land urbanization.

At the management level, the government should promote the differential urbanization development and achieve balanced urbanization development. Due to the rapid development of population urbanization, regions should control the number of the population transfer and improve the quality of the rural population. Moreover, due to the rapid development of land urbanization, regions should control the expansion of construction land and improve land use efficiency. Furthermore, the government should achieve the equalization of social public services, appropriately expand the area of urban land and build new urban areas to disperse the surplus population.

## 7. Limitation and future research

It should be noted that there are some limitations in this paper. Due to different statistical calibers, some data had different values in different statistical yearbooks and missing data was calculated by the interpolation method, resulting in a small part of the data deviating from the actual ones.

However, in the future research, the contradiction and relationship between population and land in the process of urbanization should be considered in the international context. There is no unified scientific method to measure population-land urbanization coordination and the standard for the classification of coordination degree is in disagreement, which provides the direction for future research.

## Supporting information

S1 Table Data for MDCE of population-land urbanization in the YEB from 2007 to 2017-spreadsheet. (XLS)

S2 Table Data for speed coordination evaluation of the population-land urbanization in the YEB in 2007, 2010, 2013, 2017-spreadsheet. (XLS)

S3 Table Data for level consistency evaluation of the population-land urbanization in the YEB in 2007, 2010, 2013, 2017-spreadsheet. (XLS)

S4 Table Data for space matching evaluation of the population-land urbanization in the YEB in 2007, 2010, 2013, 2017-spreadsheet. (XLS)
